# Ultrafast quantum beats of anisotropic excitons in atomically thin ReS_2_

**DOI:** 10.1038/s41467-017-02802-8

**Published:** 2018-01-24

**Authors:** Sangwan Sim, Doeon Lee, Artur V. Trifonov, Taeyoung Kim, Soonyoung Cha, Ji Ho Sung, Sungjun Cho, Wooyoung Shim, Moon-Ho Jo, Hyunyong Choi

**Affiliations:** 1 School of Electrical and Electronic Engineering, Yonsei University, Seoul, 120-749 Korea; 2 Center for Artificial Low Dimensional Electronic Systems, Institute for Basic Science (IBS), Pohang University of Science and Technology (POSTECH), 77 Cheongam-Ro, Pohang, 790-784 Korea; 3 Spin Optics Laboratory, St. Petersburg State University, St. Petersburg, 198504 Russia; 4 Division of Advanced Materials Science, Pohang University of Science and Technology (POSTECH), 77 Cheongam-Ro, Pohang, 790-784 Korea; 5 Department of Materials Science and Engineering, Yonsei University, Seoul, 120-749 Korea; 6 Department of Materials Science and Engineering, Pohang University of Science and Technology (POSTECH), 77 Cheongam-Ro, Pohang, 790-784 Korea

## Abstract

Quantum beats, periodic oscillations arising from coherent superposition states, have enabled exploration of novel coherent phenomena. Originating from strong Coulomb interactions and reduced dielectric screening, two-dimensional transition metal dichalcogenides exhibit strongly bound excitons either in a single structure or hetero-counterpart; however, quantum coherence between excitons is barely known to date. Here we observe exciton quantum beats in atomically thin ReS_2_ and further modulate the intensity of the quantum beats signal. Surprisingly, linearly polarized excitons behave like a coherently coupled three-level system exhibiting quantum beats, even though they exhibit anisotropic exciton orientations and optical selection rules. Theoretical studies are also provided to clarify that the observed quantum beats originate from pure quantum coherence, not from classical interference. Furthermore, we modulate on/off quantum beats only by laser polarization. This work provides an ideal laboratory toward polarization-controlled exciton quantum beats in two-dimensional materials.

## Abstract (web summary)

Quantum beats are periodic oscillations originating from the superposition of coherent quantum states. Here, the authors observe exciton quantum beats in the optical spectrum of atomically thin ReS_2_, and modulate the intensity of the quantum beat signal by means of light polarisation.

## Introduction

**Fig. 1:**
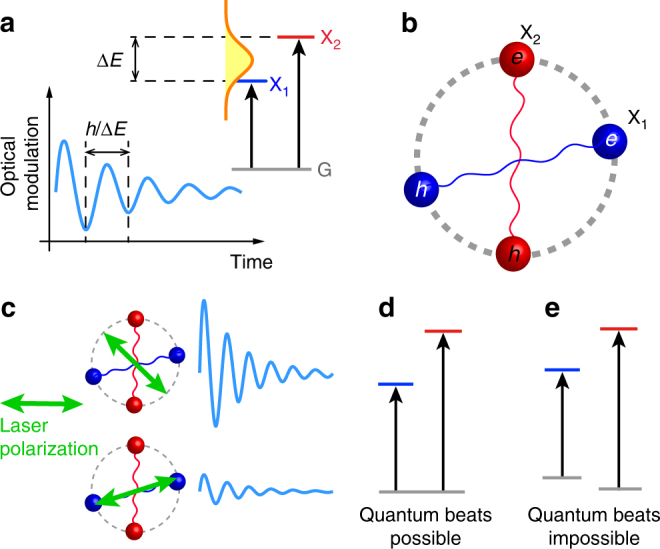
Quantum beats in ReS_2_. **a** Coherent superposition of resonantly excited two excitons (blue (X_1_) and red (X_2_) states) can lead to beating optical signals in the time domain (light blue oscillation). The oscillation period corresponds exactly to the energy different (Δ*E*) of the excited states. **b** Illustration of anisotropic excitons (dumbbell-shaped electron–hole pairs) in ReS_2_. The two lowest excitons, X_1_ and X_2_, are linearly polarized with different in-plane orientations. **c** Controlling quantum beats (light blue oscillations) of excitons by manipulating laser polarization (arrows). Blue (red) color represents X_1_ (X_2_). While the quantum beat is expected to be large when optical polarization is adjusted to excite both two excitons in a balanced way (upper schematics), it will weaken if only one of the two exciton states is excited by light asymmetrically (lower schematics). **d**, **e** Three-level system with a common state (**d**) and two independent two-level systems (**e**)

When resonantly excited quantum states have mutual coherence, their quantum superposition leads to periodically oscillating signals in optical domains (e.g., fluorescence^1^, photon echo^2,3^, and pump-probe spectroscopy^2^), where the oscillation frequency exactly matches the energy separation of the coupled states (Fig. 1a)^1,4,5^. This phenomenon, referred to as quantum beats, is of great importance for understanding the coherence of excited states in light–matter interaction because it reveals the mutual quantum coherence directly in the time domain^1,4,5^. Moreover, quantum beats play a crucial role in understanding many intriguing coherent phenomena such as lasing without population inversion^6^ and quantum entanglement^7^. This effect can also be utilized in ultrafast optoelectronic devices, including tunable terahertz emitters^8^ and optical switches^9^.

Recently, two-dimensional (2D) transition metal dichalcogenides (TMDs) have received considerable attentions due to their excellent excitonic properties, resulting from weak dielectric screening and strong quantum confinement^10–15^. To date, most of the related study has performed on MX_2_ (M = Mo, W; X = S, Se), where researchers have identified various types of excitonic coherent couplings^16–18^, such as exciton–trion^19–21^, exciton–ground^22^, and valley excitons^23–27^. For the quantum beats, however, only limited observations were reported^19–21^. The valley excitons do not exhibit quantum beats due to the energetic degeneracy. The so-called A–B excitons^28^, arising from the valence band splitting, have not shown any signature of coherence due to their rapid decoherence. Although only the exciton–trion has exhibited the quantum beats^19–21^, the presence of trions and their concentrations significantly depend on extrinsic conditions such as the doping level^29,30^. Because a trion state is a just charged version of a neutral exciton, they have same optical selection rules^23^, such that it is difficult to expect rich physics of exciton quantum beats arising from various combinations of light-polarization dependencies^7,31,32^.

Rhenium dichalcogenides are recently emerging 2D semiconductors containing a low in-plane crystal symmetry^33–36^. Interestingly, they exhibit strongly anisotropic excitons which have different orientations and optical selection rules for linearly polarized light^37–39^. Figure 1b illustrates the in-plane anisotropy of the lowest two excitons, labeled by X_1_ and X_2_, respectively, in a representative rhenium dichalcogenide ReS_2_^37^. Recent studies have shown that these anisotropic excitons hold great potentials for various optoelectronic applications, such as light-polarization-controlled switches, modulators, and optical computations^35,38–43^. Also, unlike trions and valley excitons in MX_2_, the anisotropic excitons persist regardless of the doping level and the layer number^37,38,44^. Thus, one may expect that ReS_2_ may provide an ideal platform for studying coherent phenomena in 2D semiconductors. If ReS_2_ exhibits quantum beats, the non-identical orientations of the excitons may offer new polarization-dependent phenomena, as illustrated in Fig. 1c. However, the quantum coherence of ReS_2_ remains completely elusive.

Meanwhile, there has been one critical issue on observing the exciton quantum beats. It is known that only the coherent three-level system with a common state can generate quantum beats (e.g., Fig. 1d), but uncoupled oscillators cannot (Fig. 1e)^4^. The latter can cause quantum beats-like interference whose origin does not lie on the quantum superposition. Since it is very difficult to distinguish quantum beats from the classical interference, experimental verification of the pure quantum coherence has been significantly hindered^4,45^.

In this work, by using ultrafast two-pulse pump-probe spectroscopy, we observe quantum beats in a few-layer ReS_2_ and modulate the intensity of the quantum beats signal. Our observation identifies that the phenomenon arises from quantum coherence between X_1_ and X_2_ which behave exactly as a coherently coupled three-level system, even though they have completely different orientations and optical selection rules. Our theoretical investigations prove that our two-pulse pump-probe technique does not allow the interference hindering the observation of quantum beats, unlike the three-pulse four-wave mixing experiments. Further, we modulate on/off quantum beats only by laser polarization, highlighting the anisotropic nature of the ReS_2_ excitons.

## Results

### Sample and experiment

**Fig. 2:**
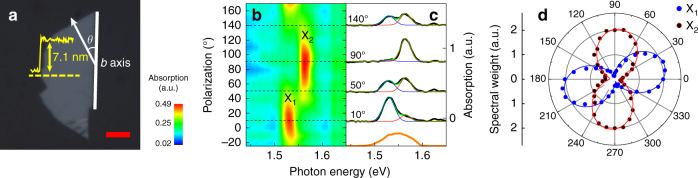
Anisotropic excitons in ReS_2_. **a** Optical image of the few-layer ReS_2_. White line along the edge of the flake represents the *b* axis. *θ* is the angle optical polarization (arrow). Yellow graph shows the AFM profile along the dashed line. The scale bar is 20 μm. **b**–**d** Polarization-dependent absorption of the ReS_2_ (**b**) and several linecuts at four different *θ* (black lines in (**c**)). The blue (red) lines are Voigt fits of the X_1_ (X_2_). Corresponding Voigt spectral weights (dots) and fits (lines, Supplementary Note 1) are shown in **d**. Orange line in **c** is the pump profile

The few-layer ReS_2_ flake (8–9 layers; Fig. 2a) was exfoliated on a sapphire substrate from bulk single crystal, using polydimethylsiloxane-stamp-assisted mechanical exfoliation technique^46^. ReS_2_ has an anisotropic crystal structure with distorted 1T phase, forming Re chains along the *b* axis (thick white line in Fig. 2a)^33,37,38^. We identify the energies of the anisotropic excitons by using static spectroscopy. Figure 2b, c shows absorption resonances of the two lowest excitons (X_1_ at 1.531 eV and X_2_ at 1.566 eV), exhibiting characteristic dependencies on the light polarization (*θ*, which measures the polarization angle with respect to the *b* axis; see Fig. 2a). Corresponding *θ*-resolved spectral weights in Fig. 2d show their anisotropic nature more clearly (Supplementary Note 1)^37,39^.

Our pump-probe experiments, based on a 250 kHz Ti:sapphire regenerative amplifier (Coherent 9050), were performed by using linearly polarized pulses centered at 800 nm (Supplementary Fig. 1). The pulse spectrum covers both X_1_ and X_2_ to excite them simultaneously (orange line in Fig. 2c). A weak portion of split beam serves as the probe pulse, which transmits the sample and reaches the detector after passing through a monochromator (Supplementary Fig. 2). The detection wavelength is set to the center of X_1_ and X_2_ (i.e., 800 nm) to maximize the beating amplitude^47^. We measure the differential transmittance (DT) as a function of pump-probe time delay (*τ*). The DT signal is defined by Δ*T*/*T*, where *T* is the probe intensity without pump, and Δ*T* is the pump-induced change in the probe intensity. Pump fluence was fixed at 15 μJ cm^−2^, and sample temperature was set at 79 K for all experiments.

### Observation of quantum beats

**Fig. 3:**
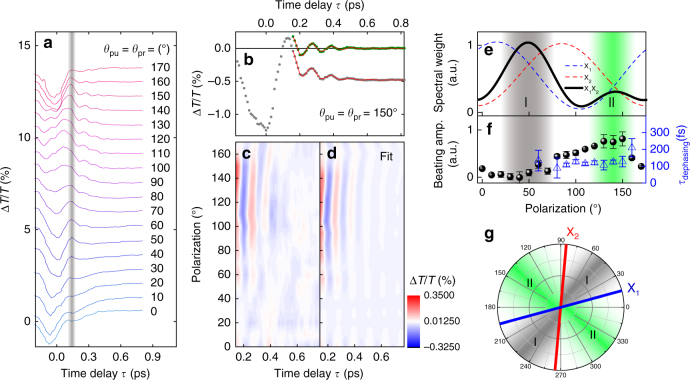
Polarization-dependent quantum beat of excitons with co-polarized pump-probe. **a** *θ* ( = *θ*_pr_ = * θ*_pu_)-resolved DT (offset for clarity). Analyses of the quantum beat are performed on the right side of the vertical gray line. **b** Original DT trace (gray dots) and its BG-subtracted version (green dots) at *θ* = 150°. Solid lines are fits. **c**, **d** Polarization-dependent BG-subtracted DT signals (**c**) and fits (**d**). **e** Dashed lines are spectral weights of X_1_ (blue) and X_2_ (red), obtained from static absorption in Fig. 2d. The black line is their product. The gray and green areas, indicated by I and II, respectively, are the regions where the theoretical beating amplitude (proportional to the black line in **e**) is expected to have local maxima (see main text). **f** *θ*-dependent beating amplitude (dots, left axis) and dephasing time constants (triangles, right axis), extracted from the fits in **d**. The error bars represent 95% confidence intervals for the fitting parameters. **g** A schematic polar diagram illustrating the regions I and II and orientations of the excitons. The blue (red) line indicates the polarization angle at which the spectral weight of X_1_ (X_2_) is maximized

We first explore the *θ*-resolved quantum beats with co-polarized pump-probe configuration (i.e., *θ* = *θ*_pu_ = *θ*_pr_, where *θ*_pu_ (*θ*_pr_) is the pump (probe) polarization). Figure 3a shows measured DT traces; we observe periodically oscillating signals after *τ* ~ 0.15 ps (right side of the gray vertical line), exhibiting strong *θ* dependence. The oscillating DT can be described by a damped harmonic oscillator ($B\exp ( { - \tau /\tau _{\mathrm{dephasing}}} )\cos \left( {\left( {2\pi /T_{\mathrm{p}}} \right)\tau + \varphi } \right)$, where *B* is the beating amplitude, $\tau _{\mathrm{dephasing}}$ is the dephasing time constant, *T*_p_ is the oscillation period, and *ϕ* is the phase) superimposed on background (BG) dynamics. Figure 3b shows a fitting example, where a DT trace at *θ* = 150° (gray dots) is well reproduced by the model (solid line), and the BG-subtracted version (green dots) indeed exhibits the behavior of a simple damped harmonic oscillator. Importantly, the oscillation period of *T*_p_ = 116 fs agrees exactly with the energy splitting between X_1_ and X_2_ (Δ*E* ~ 35 meV) via the relation $T_{\mathrm{p}} = 2\pi \hbar /\Delta E$^48^. This oscillating behavior is one apparent hallmark of the quantum coherence arising from the exciton quantum beats (note that, although the laser pulse width (~50 fs) is not very short compare to the oscillation period, it does not prohibit the observation of quantum beats; see Supplementary Fig. 3). Other evidence corroborates this assignment. First, the beatings have almost the same *T*_p_ regardless of *θ* (Fig. 3c, d), which agrees with the fact that the X_1_–X_2_ splitting has no *θ* dependence^37^. Second, no pronounced phonon mode has been reported at this energy of 35 meV in ReS_2_^49^, indicating that the oscillations do not come from the coherent phonon. In addition, no quantum beats were observed when the pump resonantly excites one of the two excitons, which further directly proves that the coherent oscillations do not originate from any single excitonic feature (Supplementary Fig. 4).

We obtained the dephasing time constant $\tau _{{\mathrm{dephasing}}}$ = 100–200 fs, as indicated by triangles in Fig. 3f, where missing values are due to significantly large uncertainty. The population relaxation is unlikely to cause this rather fast dephasing because its time constant (10–40 ps)^40^ is much longer than $\tau _{{\mathrm{dephasing}}}$. We assign the main dephasing mechanism to the inhomogeneity-induced dephasing, based on the fact that exciton absorption linewidths are dominated by the inhomogeneous broadening that originates from chalcogenide vacancy defects and other impurities (Supplementary Note 1). Thus, $\tau _{{\mathrm{dephasing}}}$ sets the lower bound of the intrinsic X_1_–X_2_ decoherence time.

The observed $\tau _{{\mathrm{dephasing}}}$ is comparable with the decoherence time of the valley excitons in monolayer MX_2_ (~100 fs at 10 K)^27^. This result is rather surprising, considering the following facts. First, unlike the energetically degenerate valley excitons, the X_1_–X_2_ dephasing dynamics should include the fast interexcitonic relaxation^16^ from the higher X_2_ to lower X_1_. Second, the experimental temperature of this work is relatively high (79 K). Therefore, our observation implies that the excitonic quantum coherence in ReS_2_ render significant robustness against relaxation dynamics and temperature variations. Further experiments, such as multidimensional^16^ and photon-echo^27^ spectroscopy, are required to accurately determine timescales of the interexcitonic relaxation and the decoherence mechanism.

**Fig. 4:**
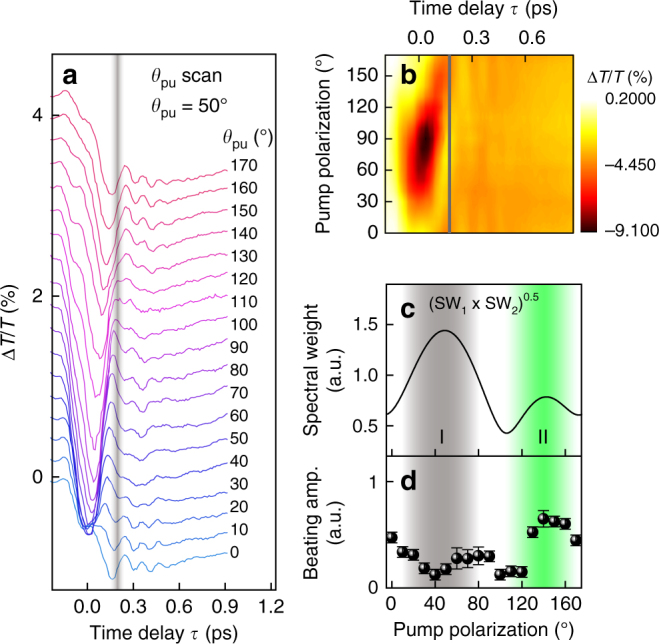
Pump-polarization-dependent quantum beats. **a**, **b** *θ*_pu_-resolved DT with offset (**a**) and its contour plot (**b**). *θ*_pr_ is set at 50°. Analyses are performed on the right side of the vertical gray line. **c**, **d** Comparison of beating amplitude obtained from theory (**c**) and experiments (**d**). In **c**, the square root of the product of the excitons’ spectral weights is shown, which is theoretically proportional to the beating amplitude (see Supplementary Note 3). Gray (green) area represents the region I (II). The error bars represent 95% confidence intervals for the fitting parameters

We should comment on peak-like and oscillating DT signals near *τ* = 0 ps and at *τ* < 0 ps (left side of the gray vertical line in Fig. 3a). Such responses can arise from optical Stark effect^38^, coherent oscillations^4^, four-wave mixed signal in the probe direction^50^, and polariton propagation^51^. Because it is very elusive to distinguish the quantum beats from these processes near *τ* = 0 ps^4^, we performed analysis limiting *τ* longer than 0.15 ps, as mentioned above. This assumption will be justified as discussed later in Fig. 4.

### Quantum beats vs. polarization interference

In this section, we theoretically confirm that the observed quantum beats originate from the pure quantum coherence. As briefly discussed in the introduction, it is known that quantum beats can be generated only in a coupled three-level system with a common state (Fig. 1d). In contrast, the independent two-level oscillators (Fig. 1e) can cause quantum beats-like signal, the so-called polarization interference (PI), arising from electromagnetic interference at the detector^4^. Because PI has the same temporal shape as the quantum beats, many researchers have devoted much effort to distinguish these phenomena since the first discovery by Lambert et al.^52^. There exist somewhat complicated methods for the distinction of quantum beats form PI, which are based on time-resolved or spectrally resolved analyses in the three-pulse four-wave-mixing experiments^45,53^. However, for the two-pulse pump-probe technique, there has been no rigorous discussion on the PI issue although it has been widely utilized to study quantum beats over the past several decades^2,48,50,54,55^.

To resolve this issue, we perform theoretical analysis based on the semi-classical Liouville–von-Neumann equation (full calculations are provided in Supplementary Note 2). The key result is that the two-pulse pump-probe technique does not allow PI, meaning no additional check processes are required to confirm the occurrence of the real quantum beats, which is different from the case of the three-pulse four-wave-mixing experiments. Thus, the two-pulse pump-probe technique is a powerful and convenient tool to identify the quantum beats. Also, our theoretical analysis further confirms that the observed X_1_–X_2_ quantum beats originate from quantum coherence in a coupled three-level configuration.

### Polarization-controlled quantum beats

We now discuss the *θ* dependence of the quantum beats. In Fig. 3c, d, the BG-subtracted DT traces show that the beating is clearly visible only at *θ* ~ 90–150°. This is corroborated by their Fourier transforms (see Supplementary Fig. 5), which show a peak near Δ*E* ~ 35 meV only at the same *θ* range. This clear *θ* dependence suggests that we can switch on/off the quantum beats simply by manipulating the laser optical polarization.

More in-depth understanding of the *θ*-dependent quantum beats can be obtained by considering a simple theoretical model. The beating amplitude *B* can be assumed to be proportional to the product of exciton spectral weight (SW), i.e., $B \propto {\mathrm{SW}}_{\mathrm{1}} \times {\mathrm{SW}}_{\mathrm{2}}$ (refs ^4,56^), where subscripts 1 and 2 mean X_1_ and X_2_, respectively. In Fig. 3e, we plot the *θ*-dependent SW_1_ and SW_2_ (dashed lines) obtained from static absorption measurements, and their product (${\mathrm{SW}}_{\mathrm{1}} \times {\mathrm{SW}}_{\mathrm{2}}$, solid black line). We see that *B* ($\propto {\mathrm{SW}}_{\mathrm{1}} \times {\mathrm{SW}}_{\mathrm{2}}$) shows peaks at *θ* ~ 50° and 140°, when SW_1_ and SW_2_ intersect. It indicates that quantum beats have relatively large intensity when the population of the two excitons is equally balanced^56^. Note that the magnitude of the two theoretical peaks is different because the polarization direction of each exciton (i.e., polarization directions at which each SW reaches its maximum, see blue and red lines in Fig. 3g) is not exactly orthogonal to each other. We refer these two peak areas to region I (gray shaded) and II (green shaded), respectively.

Let us now compare the prediction with the experimentally obtained *B* (black dots in Fig. 3f); while the measured *B* peak in the region II agrees well with the theory, a rather small value is observed in the region I. To explain the latter, we consider two possibilities. First, recent Raman spectroscopy of ReS_2_ showed that the most prominent phonon mode (mode III; ref. ^49^) has strong anisotropy, and the polarization angle at which the phonon response is maximized coincides with the region I. Thus, weak beats in this region may arise from the anisotropic exciton–phonon scattering which leads to extremely fast dephasing before the analyzed *τ* range. Second, as indicated by blue and red lines in Fig. 3g, the angle between X_1_ and X_2_ in the region I is smaller than that in the region II. Considering excitons’ anisotropic transitions follow similar polarization dependences in the momentum space^57^, electron and hole states of X_2_ can be located relatively close to those of X_1_ in the region I. This may lead to the fast dephasing through rapid relaxation from X_2_ to X_1_. More studies are needed to figure out this anomaly accurately.

So far we have discussed the polarization dependence of the quantum beats with co-polarized pump-probe configuration. Although this analysis offers meaningful physics of the anisotropic quantum coherence, further confirmation should be performed for more accurate understanding. For example, BG dynamics at *τ* > 0.15 ps in Fig. 3a show significant *θ* dependence, which may affect or mask the quantum beats signals in the DT transients. For this reason, we employed additional experiments under various pump-probe polarization configurations. Figure 4a shows DT traces measured by scanning *θ*_pu_ at a fixed *θ*_pr_ (=50°), and Fig. 4b displays its contour version (fits and Fourier spectra are provided in Supplementary Fig. 6). We can see that, unlike the case of co-polarization (Fig. 3a), BG dynamics in Fig. 4a has little dependence on *θ*_pu_ at *τ* > ~0.15 ps (right side of the vertical gray line). In contrast, the beating amplitude has similar polarization dependence as in the case of the co-polarization; the measured *B* (Fig. 4d) follows the theoretical prediction (Fig. 4c) only in the region II and show anomalously weak beating in the region I. This result suggests that the effect of BG dynamics on the quantum beat is trivial. Note that the square root of the theoretical curve ($\sqrt {{\mathrm{SW}}_{\mathrm{1}} \times {\mathrm{SW}}_{\mathrm{2}}}$) in Fig. 4c is due to the invariance of *θ*_pr_ (see Supplementary Note 3 and Supplementary Fig. 7 for the detailed discussion). Moreover, as shown in Fig. 4b, DT dips near *τ* = 0 ps exhibits the largest signal near *θ*_pu_ = 80°, which is completely different from the *θ*_pu_ dependence of *B* (Fig. 4d). This result implies that *B* does not have a significant correlation with the DT signals near *τ* = 0 ps, justifying our analysis performed at *τ* > 0.15 ps.

**Fig. 5:**
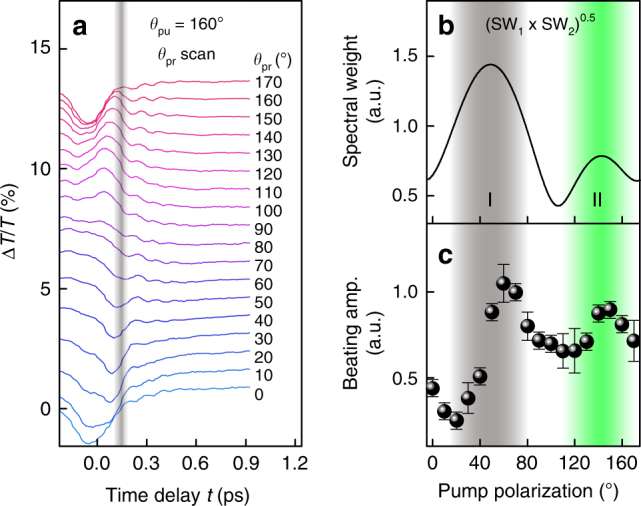
Probe-polarization-dependent quantum beats. **a** *θ*_pr_-resolved DT with offset. *θ*_pu_ is fixed at 160°. Analyses are performed on the right side of the vertical gray line. **b**, **c** Comparison of beating amplitude obtained from theory (**b**) and experiments (**c**). Solid line in **b** is same as that in Fig. 4c. Gray (green) area represents the region I (II). The error bars represent 95% confidence intervals for the fitting parameters

Finally, we examine how the exciton quantum beats change with *θ*_pr_. Here *θ*_pu_ was fixed at 160° to obtain large beating, based on the results in Fig. 3. Overall shapes of DT traces (Fig. 5a; fits and Fourier spectra are provided in Supplementary Fig. 8) closely resemble those measured in the co-polarization experiment (Fig. 3a), meaning that the polarization dependence of the BG dynamics is determined by the probe. For the beating amplitude, however, its polarization dependence is different from the case of the co-polarization experiment in Fig. 3f; the measured *B* (dots in Fig. 5c) agrees well with the theory (line in Fig. 5b) both for regions I and II, i.e., shows no anomalous quenching of the quantum beats even in the region I. This can be understood by the following way. As discussed above, the anomalously weak beating possibly originates from the fast dephasing of excitons generated in the region I. Thus, excitons generated in the region II by excitation at *θ*_pu_ = 160° are not expected to suffer such fast dephasing, agreeing with the observation in Fig. 5c. This result supports the self-consistency of our analysis.

## Discussion

In summary, we have observed quantum beats of anisotropic excitons in 2D ReS_2_. Considering that researchers have observed only beatings between excitons and trions in 2D TMDs so far, our observation is rather surprising because the anisotropic excitons show completely different optical selection rules and orientations. Despite the presence of interexcitonic relaxation and the relatively high experimental temperature, the observed dephasing time of 100–200 fs is comparable to that of valley excitons in MX_2_ at 10 K, implying strong robustness of exciton coherence in ReS_2_. Moreover, we provide theoretical analysis which proves that the two-pulse pump-probe spectroscopy measure only pure quantum beats without being hindered by the unwanted interference (PI), unlike three-pulse four-wave-mixing experiments. This analysis is expected to be a theoretical basis justifying the use of the pump-probe in future quantum beats studies. We also study polarization-resolved quantum beats, including on/off control and anomalous polarization dependence. This result highlights the characteristic anisotropy of ReS_2_ excitons. Besides, because X_1_ and X_2_ are closely located near 800 nm in the spectral domain, it is possible to generate exciton quantum beats simply by injecting the output of commercial Ti:sapphire lasers without any wavelength conversion. Therefore, we expect that our work will stimulate theoretical and experimental discovery on the exciton quantum beats in two-dimensional materials.

### Data availability

The data that support the findings of this study are available from the corresponding author upon reasonable request.

## Electronic supplementary material

Supplementary Information
