# Publisher Correction: Ultrafast quantum beats of anisotropic excitons in atomically thin ReS_2_

**DOI:** 10.1038/s41467-018-03558-5

**Published:** 2018-03-22

**Authors:** Sangwan Sim, Doeon Lee, Artur V. Trifonov, Taeyoung Kim, Soonyoung Cha, Ji Ho Sung, Sungjun Cho, Wooyoung Shim, Moon-Ho Jo, Hyunyong Choi

**Affiliations:** 1 School of Electrical and Electronic Engineering, Yonsei University, 120-749 Seoul, Korea; 2 Center for Artificial Low Dimensional Electronic Systems, Institute for Basic Science (IBS), Pohang University of Science and Technology (POSTECH), 77 Cheongam-Ro, 790-784 Pohang, Korea; 3 Spin Optics Laboratory, St. Petersburg State University, 198504 St. Petersburg, Russia; 4 Division of Advanced Materials Science, Pohang University of Science and Technology (POSTECH), 77 Cheongam-Ro, 790-784 Pohang, Korea; 5 Department of Materials Science and Engineering, Yonsei University, 120-749 Seoul, Korea; 6 Department of Materials Science and Engineering, Pohang University of Science and Technology (POSTECH), 77 Cheongam-Ro, 790-784 Pohang, Korea

Correction to: *Nature Communications* 10.1038/s41467-017-02802-8, published online 24 January 2018

The original version of this Article contained errors in Fig. 3g and Fig. 4d. In Fig. 3g, the grey shaded area was rendered incorrectly. The correct version of Fig. 3g is:

which replaces the previous incorrect version:

In Figure 4d, the black data points and error bars were omitted. The correct version of Fig. 4d is:

which replaces the previous incorrect version:

These errors have now been corrected in both the PDF and HTML versions of the Article.

