# Supplementary material for: Ultrafast quantum beats of anisotropic excitons in atomically thin ReS2
Source: Nat Commun. 2018 Jan 24;9:351. doi: 10.1038/s41467-017-02802-8 (PMC5783952; doi:10.1038/s41467-017-02802-8)
Supplement: Supplementary file 1 — Supplementary Information [file 41467_2017_2802_MOESM1_ESM.pdf]

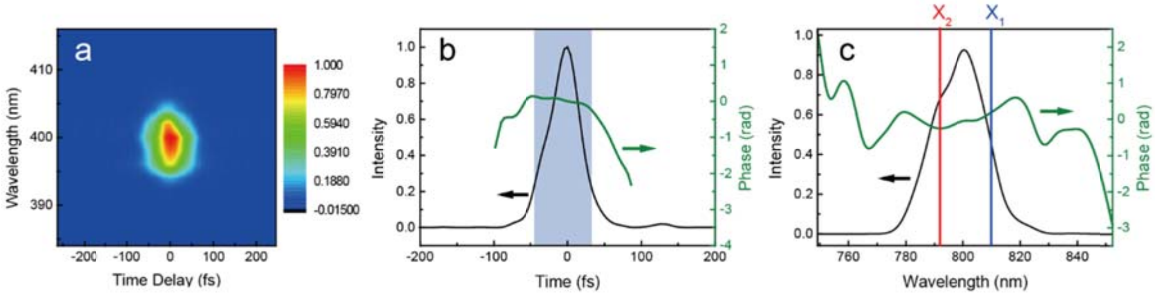


**Supplementary Figure 1 | Temporal and spectral profile of laser pulses**. **a**, Frequency-resolved optical grating (FROG) measurement of the 800 nm pulses, based on second harmonic generation in a beta barium borate crystal. **b,c,** Corresponding temporal (spectral) intensity and phase are shown in **b** (**c**), extracted by using the FROG algorithm[1] [reference link: http://frog.gatech.edu]. The FROG error is $G$ = 0.00946. In (**b**), we have identified that our pulse has ~ 50 fs pulse width (black line) and contains small quadratic phase distortion (linear positive chirp; green line) in the strong intensity region (blue shaded region). Regarding the spectral profile (**c**), the phase (green line) exhibits no significant change at the center frequencies of $X_1$ and $X_2$ (vertical red and green lines, respectively). This result indicates that the pump pulse excites $X_1$ and $X_2$ almost simultaneously in our quantum beats measurements.

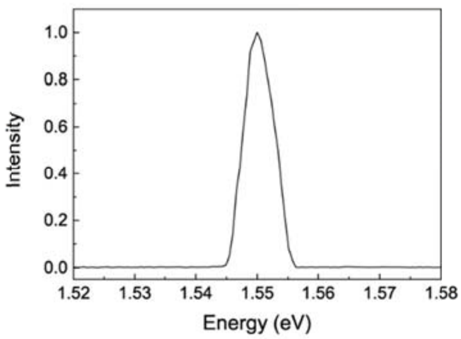


**Supplementary Figure 2 | Spectral profile of the probe pulse at detector.** As mentioned in the main text, the probe pulse transmitted through the sample passes through the monochromator and reaches the detector. The spectral width of ~ 6 meV measured at the detector (this figure) is about one-seventh of the original pulse, indicating a large broadening in the time domain. However, the increase in the temporal width has no effect on the time resolution because this broadening occurs after the probe beam passes through the sample.

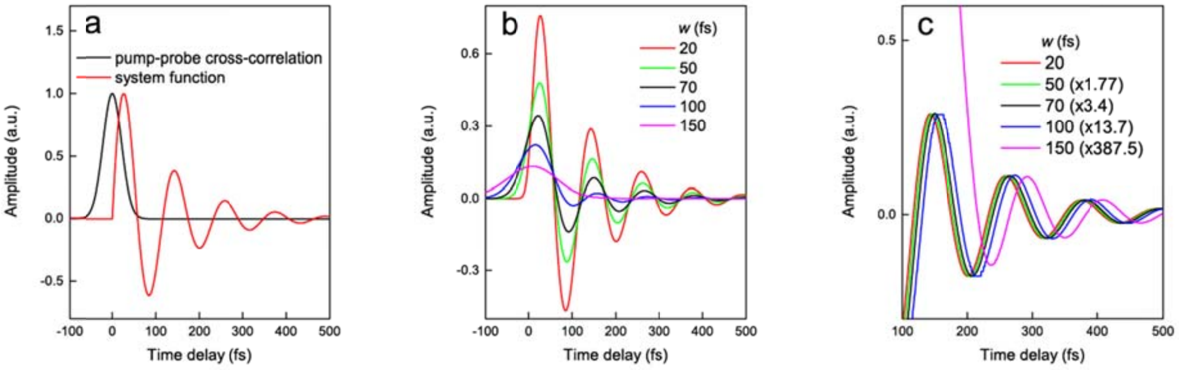


**Supplementary Figure 3 | Effect of the pulse width on DT signals. a**, DT signals can be mathematically expressed as the convolution of the pump-probe cross-correlation Gaussian (black line) and the system function (red line)[2]. Here, in order to investigate the effect of the pulse width on the quantum beat signals, we set the system function to be the damped sinusoidal function, $\Theta(\tau)\sin(2\pi\tau/T_{\mathrm{p}})\exp(-\tau/\tau_{\mathrm{dephasing}})$; $\Theta(t)$ is the Heaviside step function, the oscillation period ($T_{\mathrm{p}}$) and the dephasing time ($\tau_{\mathrm{dephasing}}$) are set to 116 fs and 120 fs, respectively, where the parameters are based on our measurements shown in the main text (Fig. 3). **b**, Calculated convolutions of the system function and the pump-probe cross-correlation Gaussians for five different Gaussian widths (*w*). The oscillation amplitude decreases with increasing *w*, indicating that shorter pulse width is favorable for obtaining better signal-to-noise ratio. Importantly, we can see that *w* ~ 70 fs of our laser pulse is capable of measuring quantum beats (see black line). This result further justifies our experiments. **c**, Curves in (**b**) are re-plotted with scaled by factors in parentheses. Although the oscillation phase slightly shifts and the amplitude becomes smaller with increasing *w*, all simulated curves yield oscillations with a fixed period of 116 fs. However, as shown by the magenta line (*w* = 150 fs), one loses information of quantum beats at early time-delays (< 200 fs in this case) when *w* exceeds the oscillation period.

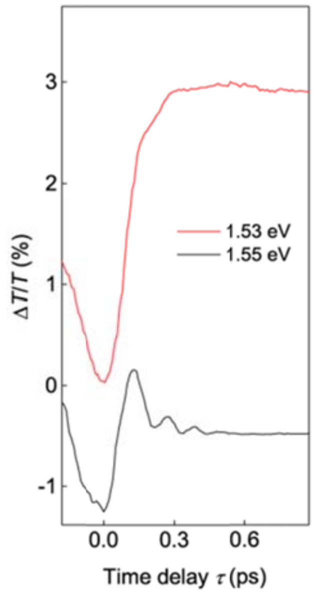


**Supplementary Figure 4 | Pump energy-dependent quantum beats.** Black (red) line is the DT trace with the pump energy of 1.55 eV (~ 1.53 eV) at $\theta_{pr}$ = $\theta_{pu}$ = 150°. Offset for clarity. When pump excites both $X_1$ and $X_2$, their coherence leads to the quantum beats shown by the black line (same as Fig. 3b in the main text). In contrast, we observe an extremely small beats when the pump dominantly excites $X_1$ (red line). This result confirms that the beatings originate from the coherence of $X_1$ and $X_2$. The negative DT dip near $\tau$ = 0 ps persists irrespective of the quantum beats, supporting our assumption in the main text. The large background of the red trace at positive time delays may result from efficient bleaching of the $X_1$ transition because the pump energy is very close to that of $X_1$.

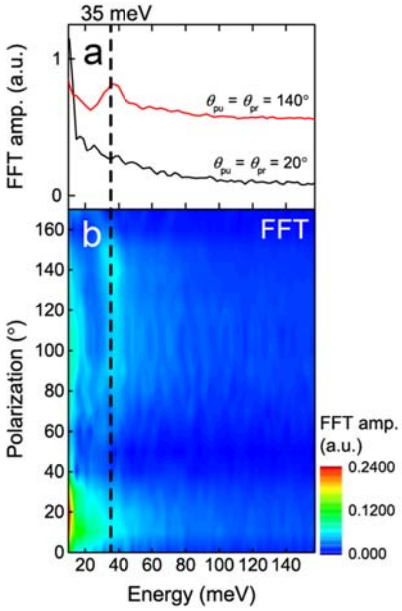


**Supplementary Figure 5 | Analysis of polarization-dependent quantum beats with co-polarized pump-probe configuration. a**,**b**, Polarization-dependent Fourier spectra of DT traces on the right side of the gray line in Fig. 3a in main text (**b**), and corresponding linecuts at two different angles (**a**). A vertical dashed line shows that Fourier profiles at $\theta$ (= $\theta_{pr}$ = $\theta_{pu}$) = 80-160° have peaks near 35 meV, which agrees with the $X_1$-$X_2$ energy separation.

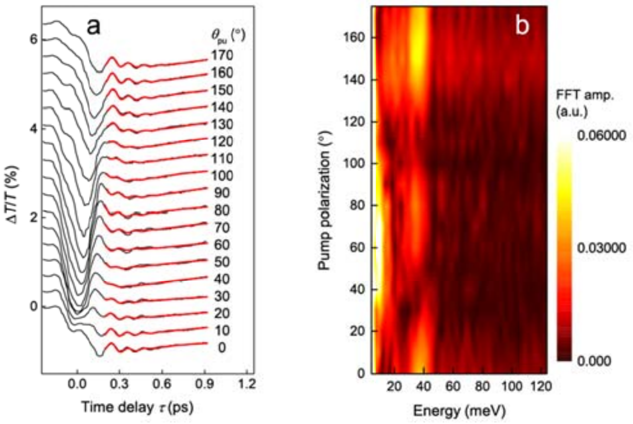


**Supplementary Figure 6 | Analysis of pump-polarization-dependent quantum beats. a**, Black lines are the same DT traces in Fig. 4a in the main text. Red lines are the corresponding fits. **b,** Corresponding Fourier spectra are shown.

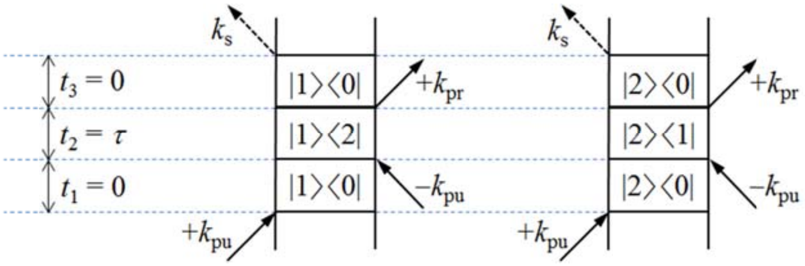


**Supplementary Figure 7 | Feynman diagrams for quantum beats**[3,4]**.** $|1\rangle$ , $|2\rangle$ and $|0\rangle$ represent $X_1$, $X_2$ and the ground state, respectively. Contributions from complex conjugates can be obtained by exchanging the bra and the ket. $k_{pu}$ ($k_{pr}$) is the pump (probe) wave vector, and $k_s = +k_{pu} - k_{pu} + k_{pr} = k_{pr}$ .

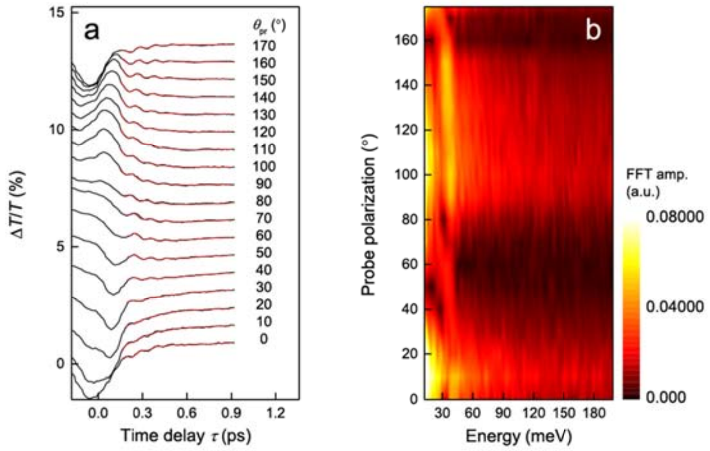


**Supplementary Figure 8 | Analysis of probe-polarization-dependent quantum beats. a**, Black lines are the same data in Fig. 5a in the main text. Red lines are fits. **b,** Corresponding Fourier spectra are shown.

**Supplementary Note 1. Analysis of static absorption spectra**

To understand broadening of excitons, we use Voigt functions to fit the static absorption of the excitons[3]. Because the Voigt function is the convolution of the Lorentzian and Gaussian function, this analysis enables us to estimate how much the homogeneous linewidth (Γ) and the inhomogeneous linewidth (σ) contribute to the total broadening of exciton resonances, respectively. Figure 2c in the main text shows such examples, where black lines are background-subtracted absorption, blue and red lines are Voigt fit lines to the $X_1$ and $X_2$, respectively, and green lines are their sums. Here, each exciton peak is fit by a built-in Voigt function in OriginPro 9.0 (function name: nlf_voigt). Through this process, we obtain the $\theta$-resolved Voigt spectral weights of $X_1$ and $X_2$, as shown by dots in Fig. 2d in the main text. They match very well with a function $a_i + b_i \cos^2(\theta - \theta_i)$ (solid lines in Fig. 2d in the main text), where the subscript $i$ = 1, 2 means $X_1$ and $X_2$, respectively, $a_i$ and $b_i$ are constants, and $\theta_i$ is the polarization angle at with the spectral weight for $X_i$ is maximized. $\theta_1$ and $\theta_2$ obtained from fits are 15° and 85°, respectively, in good agreement with previous reports[5,6~~4,5~~].

For the inhomogeneous broadening, $\sigma_1$ ($\sigma_2$) is ~ 28 (25) meV, irrespective of the optical polarization[54]. In contrast, homogeneous linewidths ($\Gamma_1$ and $\Gamma_2$) reported by the fitting software are at least one order less than the inhomogeneous linewidths, and show extremely large error ranges due to their relatively small magnitudes. It means that the exciton linewidths is governed by the inhomogeneous broadenings, in good agreement with the previous study on atomically-thin TMDs[7]. Therefore, we attributed the fast dephasing of 100-200 fs in the $ReS_2$ (Fig. 3f in the main text) to the inhomogeneous broadening-induced phase-breaking. However, further time-resolved experiments, such as ultrafast photon echo spectroscopy, is required to understand the dephasing mechanism more accurately in the

dynamic regime.

## Supplementary Note 2. Theoretical comparison of quantum beats and polarization interference in the pump-probe signal

Quantum beats-type oscillating signals can arise from i) coherent superposition of wavefunctions in the three-level system with a common state (Fig. 1d in the main text) and ii) the so-called polarization interference (PI) of two independent two-level transitions (Fig. 1e in the main text)[8]. While the former is the true quantum mechanical phenomenon, the latter is a kind of artifact that occurs at the detector. In four-wave-mixing experiments of quantum beats, special methods such as time-resolved or spectrally-resolved analyses are required to distinguish the true quantum beats from PI[8–10]. However, to the best of our knowledge, there has been no theoretical analysis of the PI issue in pump-probe measurements.

In the following, we theoretically investigate pump-probe signals in a three-level system and two independent two-level systems, respectively. We show that while pump-probe signals can arise from quantum beats in the former, does not allow PI in the latter. This result confirms that our observation originates from quantum beats in the three-level configuration of $ReS_2$ excitons.

First, let us describe the beat signals in pump-probe experiments for the case of a 3-level system. The basis of the model is the Liouville-von-Neumann equation, which in the interaction picture for a density matrix, $\rho$, reads[8]:

$$\mathrm{i}\hbar \frac{d\rho}{dt} = [V, \rho]. \tag{1}$$

Perturbation operator $V$ describes the interaction of excitons with the electromagnetic wave of a laser pulse:

$$V = \begin{pmatrix} 0 & -d_1 E(t) & -d_2 E(t) \\ -d_1^* E^*(t) & 0 & 0 \\ -d_2^* E^*(t) & 0 & 0 \end{pmatrix}. \tag{2}$$

The matrix of this operator is written in the basis of wave functions $|i\rangle$ with $i = 0$, 1, 2. Here $|0\rangle$ denotes the ground (vacuum) state of the quantum system, $|1\rangle$ and $|2\rangle$ denote the dipole active excitonic states. Operators $d_1$ and $d_2$ in Eq. (2) are the dipole moments of the interband optical transitions to excitonic states $|1\rangle$ and $|2\rangle$, $E(t)$ is the envelope of the electric field in the pulse, and the rotating wave approximation is used. The pulse is considered to be short, correspondingly, its spectral width is wide enough to cover the both optical transitions in the 3-level exciton system. No intraband optical transitions are considered that is $V_{12} = V_{21} = 0$.

A detailed analysis of density matrix evolution after the pump and probe pulses action is described in Suppl. Materials of Ref. 11. In the second order of perturbation theory, before arriving of the second (probe) pulse at time $\tau$ after the first (pump) pulse, the density matrix elements relax to the values (see Eq. (6) in Suppl. Materials of Ref. 11):

$$\rho_{0j}^{(02)}(\tau) \approx -\mathrm{i}\frac{d_j^* S_{pu}}{\hbar}\exp\left(-\tau/T_{0j}\right), \tag{3}$$

$$\rho_{jj}^{(02)}(\tau) \approx \frac{2|d_j|^2 S_{pu}^2}{\hbar^2}\exp\left(-\tau/T_{jj}\right),$$

$$\rho_{kj}^{(02)}(\tau) \approx \frac{2 d_j d_k^* S_{pu}^2}{\hbar^2}\exp\left[\mathrm{i}(\omega_k - \omega_j)\tau - \tau/T_{kj}\right],$$

$$\rho_{00}^{(02)}(\tau) = 1 - \sum_j 2|d_j|^2 |S_{pu}|^2 \exp\left(-\tau/T_{jj}\right).$$

Here $S_{pu} = E_{pu}\tau_{pu}$ is the area of the pump pulse, which is considered to have a rectangular shape; $\omega_j$ are the frequencies of optical transitions to exciton states $j=1$ and 2; $T_{ij}$ are the phenomenological times, describing the relaxation of different density matrix elements.

After the action of the second (probe) pulse, in the first order of smallness, the density matrix elements required to describe the optical polarization read:

$$\rho_{01} \propto \frac{\mathrm{i}S_{pr}}{\hbar}\left[d_1^* e^{\mathrm{i}\delta_1\tau_{pr}}\rho_{11}^{(02)} + d_2^* e^{\mathrm{i}\delta_2\tau_{pr}}\rho_{21}^{(02)} + d_1^* e^{\mathrm{i}\delta_1\tau_{pr}}\left(\rho_{11}^{(02)} - \rho_{00}^{(02)}\right)\right] \quad (4)$$

$$\rho_{02} \propto \frac{\mathrm{i}S_{pr}}{\hbar}\left[d_1^* e^{\mathrm{i}\delta_1\tau_{pr}}\rho_{12}^{(02)} + d_2^* e^{\mathrm{i}\delta_2\tau_{pr}}\rho_{22}^{(02)} + d_2^* e^{\mathrm{i}\delta_1\tau_{pr}}\left(\rho_{22}^{(02)} - \rho_{00}^{(02)}\right)\right]$$

Here $S_{pr}$ is the area of the probe pulse of duration $\tau_{pr}$, $\delta_j = \omega - \omega_j$, where $\omega$ is the optical frequency of the electromagnetic field.

The radiation of a sample with the 3-level system propagating in the direction of the transmitted probe beam in the pump-probe experiment is determined by polarization

$$P_{3lvl}(\tau,t) \propto d_1\rho_{01}^{(12)}(\tau)e^{-\mathrm{i}\omega_1 t} + d_2\rho_{02}^{(12)}(\tau)e^{-\mathrm{i}\omega_2 t} \quad (5)$$

$$\approx \frac{\mathrm{i}S_{pr}}{\hbar}\times\left[\frac{2|d_1|^2}{\hbar}\rho_{11}^{(02)}(\tau)e^{-\mathrm{i}\omega_1 t} + \frac{2|d_2|^2}{\hbar}\rho_{22}^{(02)}(\tau)e^{-\mathrm{i}\omega_2 t}\right.$$

$$-|d_1|^2 e^{-\mathrm{i}\omega_1 t}\rho_{00}^{(02)}(\tau) - |d_2|^2 e^{-\mathrm{i}\omega_2 t}\rho_{00}^{(02)}(\tau)$$

$$\left. + d_1 d_2^*\rho_{21}^{(02)}(\tau)e^{-\mathrm{i}\omega_1 t} + d_2 d_1^*\rho_{12}^{(02)}(\tau)e^{-\mathrm{i}\omega_2 t}\right]$$

Here for simplicity we have used the approximation: $e^{\mathrm{i}\delta_1\tau_{pr}} \approx 1$ and $e^{\mathrm{i}\delta_1\tau_{pr}} \approx 1$, which is valid if the duration of probe pulse is much smaller than the beating period.

As one can see from Eqs. (3, 5), the dependence on delay $\tau$ between the pump and probe

pulses is present in the density matrix elements: $\rho_{00}^{(02)}(\tau)$, $\rho_{11}^{(02)}(\tau)$, $\rho_{22}^{(02)}(\tau)$, $\rho_{12}^{(02)}(\tau)$, and $\rho_{21}^{(02)}(\tau)$. However, only the matrix elements $\rho_{12}^{(02)}(\tau)$ and $\rho_{21}^{(02)}(\tau)$ are the oscillating functions of delay $\tau$ when $\omega_1 \neq \omega_2$, see the third line in Eq. (3).

The experimentally measured signal is integrated over real time $t$ by a photodetector, $I(\tau) = \int | P_{3lv}(\tau, t) |^2 \, dt$. The integration washes out the interference beating in the real time (not in the delay time). However, the beating component in the delay dependence of the signal is conserved because $\rho_{12}^{(02)}$ and $\rho_{21}^{(02)}$ depend on $\tau$. This fact explains the quantum beats observed experimentally in the pump-probe experiments for three-level systems.

Next, let us describe the case of the two independent 2-level systems. We consider four basic states, namely, $|0\rangle$ and $|1\rangle$ are the ground state and the dipole active exciton state, respectively, for the first 2-level system; $|2\rangle$ and $|3\rangle$ are the dipole active excitonic state and the ground state, respective, for the second 2-level system. So, the dipole active excitonic states are denoted by indexes 1 and 2, as in the case of the 3-level system discussed above.

The perturbation operator $V$ describing the interaction of the quantum systems with an optical pulse reads in this case:

$$V = \begin{pmatrix} 0 & -d_1 E(t) & 0 & 0 \\ -d_1^* E^*(t) & 0 & 0 & 0 \\ 0 & 0 & 0 & -d_2 E(t) \\ 0 & 0 & -d_2^* E^*(t) & 0 \end{pmatrix} \qquad (6)$$

The expressions describing the time evolution of elements of the density matrix are easily obtained by a similar way considered above for the 3-level system. Before arriving of the probe pulse at time $\tau$ after the pump pulse, the density matrix elements

relax to the values:

$$\rho_{11}^{(02)} \approx \frac{2|d_1|^2|S_{pu}|^2}{\hbar^2}\exp\left(-\tau/T_{11}\right), \tag{7}$$

$$\rho_{22}^{(02)} \approx \frac{2|d_2|^2|S_{pu}|^2}{\hbar^2}\exp\left(-\tau/T_{22}\right),$$

$$\rho_{00}^{(02)} = 1-\frac{2|d_1|^2|S_{pu}|^2}{\hbar^2}\exp\left(-\tau/T_{11}\right),$$

$$\rho_{33}^{(02)} = 1-\frac{2|d_2|^2|S_{pu}|^2}{\hbar^2}\exp\left(-\tau/T_{22}\right).$$

After the action of the probe pulse, in the first order of smallness, the required density matrix elements are:

$$\rho_{01}^{(12)} \approx d_1^* e^{i\delta_1\tau_{pr}}\rho_{11}^{(02)} + d_1^* e^{i\delta_1\tau_{pr}}\left(\rho_{11}^{(02)} - \rho_{00}^{(02)}\right), \tag{8}$$

$$\rho_{23}^{(12)} \approx d_2^* e^{i\delta_2\tau_{pr}}\rho_{22}^{(02)} + d_2^* e^{i\delta_1\tau_{pr}}\left(\rho_{22}^{(02)} - \rho_{33}^{(02)}\right).$$

Respective polarization created by the two pulses in the two 2-level systems is described by expression:

$$P_{2lvl}(\tau,t) \propto d_1\rho_{01}^{(12)}(\tau)e^{-i\omega_1 t} + d_2\rho_{23}^{(12)}(\tau)e^{-i\omega_2 t} \tag{9}$$

$$\approx \frac{-iS_{pr}}{\hbar}\times\Big[2|d_1|^2\ \rho_{11}^{(02)}(\tau)e^{-i\omega_1 t} + 2|d_2|^2\ \rho_{22}^{(02)}e^{-i\omega_2 t}$$

$$-|d_1|^2\ e^{-i\omega_1 t}\rho_{00}^{(02)} - |d_2|^2\ e^{-i\omega_2 t}\rho_{33}^{(02)}\Big]$$

Here we have again used the approximation: $e^{i\delta_1\tau_{pr}} \approx 1$ and $e^{i\delta_1\tau_{pr}} \approx 1$. One can easily see that the dependence on delay $\tau$ is only present in the diagonal matrix elements, $\rho_{11}^{02}(\tau)$ and $\rho_{11}^{02}(\tau)$, exponentially decaying without oscillations, see Eqs. (7). The experimentally

detected signal, $I(\tau) = \int |P_{2lvl}(\tau)|^2\, dt$, is characterized by a smoothly decaying function on delay $\tau$ without any oscillations.

The diagonal matrix elements of density matrix are proportional to the occupations the corresponding states. Occupation have no any phase properties and signal proportional only to the diagonal density matrix elements (as in the case of 2 independent 2-level systems) have no any oscillations, but only exponential decay. For the three level system, as one can see from the last line of Eq. (5), the optical response includes also non diagonal elements in contrast to the two independent 2-level systems (see Eq. (9)). So the oscillations in pump-probe-signal in the limit of perturbative pulses powers is a forward confirmation of a quantum beats.

**Supplementary Note 3. Laser-polarization-dependence of quantum beats**

When the pump pulse simultaneously excited two closely located states (e.g., $X_1$ and $X_2$ in $ReS_2$) in the two-pulse pump-probe geometry, the surviving coherent processes under the rotating wave approximation and the phase matching condition are the stimulated emission, the bleach and the quantum beats[3,4]. Among them, we focus on the quantum beats, whose Feynman diagrams are shown in Supplementary Fig. 8.

The time-ordered response function corresponding to the first diagram in Supplementary Fig. 8 is given by[12,13]

$$R(t_1,t_2,t_3) = R(0,\tau,0) = \left(\frac{1}{i\hbar}\right)^3 \left(\vec{\mu}_{10}\cdot\hat{\varepsilon}_{\mathrm{pr}}\right)\left(\vec{\mu}_{02}\cdot\hat{\varepsilon}_{\mathrm{pr}}\right)\left(\vec{\mu}_{20}\cdot\hat{\varepsilon}_{\mathrm{pu}}\right)\left(\vec{\mu}_{01}\cdot\hat{\varepsilon}_{\mathrm{pu}}\right)\Theta(\tau)\exp(-\Gamma\tau)\exp(-i\omega_{21}\tau)\rho_{00}, \qquad (10)$$

where $\tau$ is the pump-probe time delay, $\vec{\mu}_{mn} = \langle m|\hat{\mu}|n\rangle$ is the transition dipole moment element

(which are assumed to be real), $\hat{\varepsilon}_{\rm pu}(\hat{\varepsilon}_{\rm pr})$ is the unit electric polarization vector of the pump (probe), $\Theta(\tau)$ is the Heaviside step function and $\rho_{00} = \langle 0|\hat{\rho}|0\rangle$ is the density matrix element. The second Feynman diagram in Supplementary Fig. 8 can be represented in a similar way. The Eq. (10) is usually considered the product of the geometrical part $((\vec{\mu}_{01}\cdot\hat{\varepsilon}_{\rm pr})(\vec{\mu}_{02}\cdot\hat{\varepsilon}_{\rm pr})(\vec{\mu}_{02}\cdot\hat{\varepsilon}_{\rm pu})(\vec{\mu}_{01}\cdot\hat{\varepsilon}_{\rm pu}))$ and the dynamical part $(\Theta(\tau)\exp(-\Gamma\tau)\exp(-i\omega_{21}\tau))$ (ref. 12). Here the geometrical part is of particular importance to anisotropic materials such as $ReS_2$ because it reflects the relative angles between the anisotropic transition dipole moment ($\vec{\mu}_{mn}$) and the light polarization ($\hat{\varepsilon}$). We now can obtain the light-polarization dependence of the beating amplitude by considering the geometrical part as follows.

If excitonic transitions from ground state were perfectly anisotropic, one can assume that

$$|\vec{\mu}_{01}\cdot\vec{\varepsilon}_{\rm pu,\,pr}| = \mu_{01}\,|\cos(\theta_{\rm pu,pr}-\theta_1)|, \quad (11)$$

$$|\vec{\mu}_{02}\cdot\vec{\varepsilon}_{\rm pu,\,pr}| = \mu_{02}\,|\cos(\theta_{\rm pu,pr}-\theta_2)|. \quad (12)$$

In this formalism, the optical transitions reach their maximum when the light polarization coincides with the orientation of each exciton (i.e., $\theta_{\rm pu,pr} \,//\, \theta_{1,2}$), and disappears completely for the perpendicular configurations (i.e., $\theta_{\rm pu,pr} \perp \theta_{1,2}$ ). However, this is inconsistent with the observed results, as the excitonic transition probability does not vanish even though the optical polarization is perpendicular to the axis of each exciton[5] (Fig. 2d in the main text). To reflect more realistic trend, by considering the relation: $\mu^2 \propto$ (oscillator strength) $\propto$ (SW), we make following phenomenological assumption,

$$|\vec{\mu}_{01}\cdot\vec{\varepsilon}_{\rm pu,\,pr}| \propto \sqrt{\mathrm{SW}_1(\theta_{\rm pu,pr})} = \sqrt{a_1 + b_1\cos^2(\theta_{\rm pu,pr}-\theta_1)}\,, \quad (13)$$

$$|\vec{\mu}_{02}\cdot\vec{\varepsilon}_{\mathrm{pu,pr}}| \propto \sqrt{\mathrm{SW}_2(\theta_{\mathrm{pu,pr}})} = \sqrt{a_2 + b_2\cos^2(\theta_{\mathrm{pu,pr}} - \theta_2)}\,, \tag{14}$$

where the spectral weigh parameters ($a_1$, $b_1$, $\theta_1$, $a_2$, $b_2$, $\theta_2$) are obtained from fitting the static absorption (see Supplementary Section 1). Thus, the magnitude of the geometrical part is proportional to the square root of product of four spectral weight functions,

$$|(\vec{\mu}_{01}\cdot\hat{\varepsilon}_{\mathrm{pr}})(\vec{\mu}_{02}\cdot\hat{\varepsilon}_{\mathrm{pr}})(\vec{\mu}_{01}\cdot\hat{\varepsilon}_{\mathrm{pu}})(\vec{\mu}_{02}\cdot\hat{\varepsilon}_{\mathrm{pu}})| \propto \sqrt{[\mathrm{SW}_1(\theta_{\mathrm{pr}})][\mathrm{SW}_2(\theta_{\mathrm{pr}})][\mathrm{SW}_1(\theta_{\mathrm{pu}})][\mathrm{SW}_2(\theta_{\mathrm{pu}})]}\,. \tag{15}$$

This result is also true for other quantum beats pathways (i.e., the second pathway in Supplementary Fig. 8 and conjugate pathways). The pump-probe signal is proportional to the sum of response functions of these pathways (i.e., $\Delta T \propto \sum R(0,\tau,0)$). Therefore, considering only the geometrical part, the relationship between the beating amplitude and pump/probe polarizations is given by

$$B \propto \sqrt{[\mathrm{SW}_1(\theta_{\mathrm{pr}})][\mathrm{SW}_2(\theta_{\mathrm{pr}})][\mathrm{SW}_1(\theta_{\mathrm{pu}})][\mathrm{SW}_2(\theta_{\mathrm{pu}})]}\,. \tag{16}$$

This result changes its form depending on the pump-probe polarization configuration. In the co-polarized pump-probe configuration (i.e., $\theta = \theta_{\mathrm{pr}} = \theta_{\mathrm{pu}}$), Eq. (16) becomes $B \propto [\mathrm{SW}_1(\theta)][\mathrm{SW}_2(\theta)]$, which is shown by the black solid line in Fig. 3e in the main text. However, when probe (pump) polarization is kept at a fixed value, $B$ in Eq. (16) should be proportional to $\sqrt{[\mathrm{SW}_1(\theta_{\mathrm{pu}})][\mathrm{SW}_2(\theta_{\mathrm{pu}})]}$ ($\sqrt{[\mathrm{SW}_1(\theta_{\mathrm{pr}})][\mathrm{SW}_2(\theta_{\mathrm{pr}})]}$), as shown in Fig. 4c (Fig. 5b) in the main text.

**Supplementary References**


1. Trebino, R. *Frequency-resolved optical gating: The measurement of ultrashort laser pulses*. (Science & Business Media, 2012).

2. Prasankumar, R. P. & Taylor, A. J. *Optical techniques for solid-state materials characterization*. (CRC Press, 2011).

3. Mukamel, S. *Principles of nonlinear optical spectroscopy*. (Oxford University Press, 1995).

4. Hamm, P. *Principles of nonlinear optical spectroscopy: a practical approach or: Mukamel for dummies*. (University of Zurich, 2005).

5. Aslan, O. B., Chenet, D. a., van der Zande, A. M., Hone, J. C. & Heinz, T. F. Linearly polarized excitons in single- and few-layer $ReS_2$ crystals. *ACS Photonics* **3,** 96–101 (2016).

6. Sim, S. *et al.* Selectively tunable optical Stark effect of anisotropic excitons in atomically thin $ReS_2$. *Nat. Commun.* **7,** 13569 (2016).

7. Moody, G. *et al.* Intrinsic homogeneous linewidth and broadening mechanisms of excitons in monolayer transition metal dichalcogenides. *Nat. Commun.* **6,** 8315 (2015).

8. Shah, J. *Ultrafast spectroscopy of semiconductors and semiconductor nanostructures*. (Springer Berlin Heidelberg).

9. Koch, M. *et al.* Quantum beats versus polarization interference: An experimental distinction. *Phys. Rev. Lett.* **69,** 3631–3634 (1992).

10. Lyssenko, V. G. *et al.* Nature of nonlinear four-wave-mixing beats in semiconductors. *Phys. Rev. B* **48,** 5720–5723 (1993).

11. Trifonov, A. V. *et al.* Multiple-frequency quantum beats of quantum confined exciton states. *Phys. Rev. B* **92,** 201301 (2015).

12. Qian, W. & Jonas, D. M. Role of cyclic sets of transition dipoles in the pump-probe polarization anisotropy: Application to square symmetric molecules and perpendicular chromophore pairs. *J. Chem. Phys.* **119,** 1611–1622 (2003).

13. Farrow, D. A., Smith, E. R., Qian, W. & Jonas, D. M. The polarization anisotropy of vibrational quantum beats in resonant pump-probe experiments: Diagrammatic calculations for square symmetric molecules. *J. Chem. Phys.* **129,** 174509 (2008).
